# New Gastropod Vectors and Tetrodotoxin Potential Expansion in Temperate Waters of the Atlantic Ocean

**DOI:** 10.3390/md10040712

**Published:** 2012-03-26

**Authors:** Marisa Silva, Joana Azevedo, Paula Rodriguez, Amparo Alfonso, Luis M. Botana, Vítor Vasconcelos

**Affiliations:** 1 Department of Biology, Faculty of Sciences, University of Porto, Rua do Campo Alegre, 4619-007 Porto, Portugal; Email: marisasilva17@gmail.com (M.S.); joana_passo@hotmail.com (J.A.); 2 Center of Marine and Environmental Research–CIMAR/CIIMAR, University of Porto, Rua dos Bragas, 289, 4050-123 Porto, Portugal; 3 Department of Chemical and Biomolecular Sciences, School of Health and Technology of Porto, Vila Nova de Gaia, 4400-330 Vila Nova de Gaia, Portugal; 4 Department of Pharmacology, Faculty of Veterinary, University of Santiago of Compostela, 27002 Lugo, Spain; Email: paula.rodriguez17@rai.usc.es (P.R.); amparo.alfonso@usc.es (A.A.); Luis.Botana@usc.es (L.M.B.)

**Keywords:** tetrodotoxin, new vectors, gastropods, North Atlantic Waters

## Abstract

Tetrodotoxin is a potent low weight marine toxin found in warm waters, especially of the Indian and Pacific Oceans. Intoxications are usually linked to the consumption of the puffer fish, although TTX was already detected in several different edible taxa. Benthic organisms such as mollusks and echinoderms, with different feeding habits, were collected monthly along the Portuguese coast from the summer of 2009 until the end of 2010. The extraction and analysis techniques were optimized and TTX and some analogues were detected for the first time in two intertidal gastropod species—*Gibbula umbilicalis* and *Monodonta lineata* by LC-MS/MS and UPLC-MS/MS. Although the levels are low, these findings suggest that monitoring of TTX and analogues in North Atlantic species should be implemented so as to detect potentially new toxin vectors and seasonal and/or geographical patterns.

## 1. Introduction

Tetrodotoxin (TTX) is a low weight potent neurotoxin, named after the Tetradontidae fish family from where it was first isolated in 1909 by Tahara and Hirata [1]. TTX is an interesting toxin, since it was reported in several taxa genetically not close related; from bacteria; marine invertebrates; terrestrial and marine vertebrates [2]. Neither its biochemical path nor its true origin is fully clarified, since three hypotheses point to its origin: endogenous [3,4], through food-chain [5,6,7,8] or through symbionts [9,10,11,12]. 

TTX is an extremely potent toxin, it binds specifically to site 1 of the voltage-gated sodium channels (Nav), occluding the external pore blocking the cellular communication and causing death by cardio-respiratory paralysis [13,14,15,16]. Several poisoning incidents have occurred, especially in Asia, with Japan being the most affected country and where Fugu is considered a delicacy. Japan is the only country to have guideline values for TTX [17]. Although TTX-bearers are typical of warm waters, recent studies report the possible migration of these toxic species from the Red Sea to the Mediterranean Sea through the Suez Canal [18,19,20]. This may happen due the opening of new corridors allied to the increase of water temperature as a result of climate change. These factors all together probably influenced the bidirectional migration of species between the Red Sea and the Mediterranean Sea, resulting in the increase of poisoning incidents, especially due to the ingestion of toxic alien species, among them TTX-bearers [18,19,20,21,22]. A good example of intoxication incidents caused by TTX-bearers was the ingestion of the elongated puffer, *Lagocephalus sceleratus*. Its presence in the Mediterranean was first reported in 2003, causing several poisoning incidents in Egypt in the end of 2004 and in Israel between 2005 and 2008. Fortunately all patients recovered [18,20,23].

In October 2007 a case of TTX poisoning occurred in Malaga, Spain, due to the ingestion of a specimen of *Charonia lampas*, an autochthonous predatory gastropod from Atlantic and Mediterranean waters, caught in the southern Portuguese waters [21]. This episode was the first report TTX occurrence in autochthonous species in Atlantic and Mediterranean waters and triggered our investigation to monitor different invertebrate species in several sites of our coast [21]. 

In this work our goal was to detect the presence of TTX and some analogues in several marine invertebrate species collected along the continental Portuguese coast, by using UPLC-MS/MS and LC-MS/MS.

## 2. Results and Discussion

**Figure 1:**
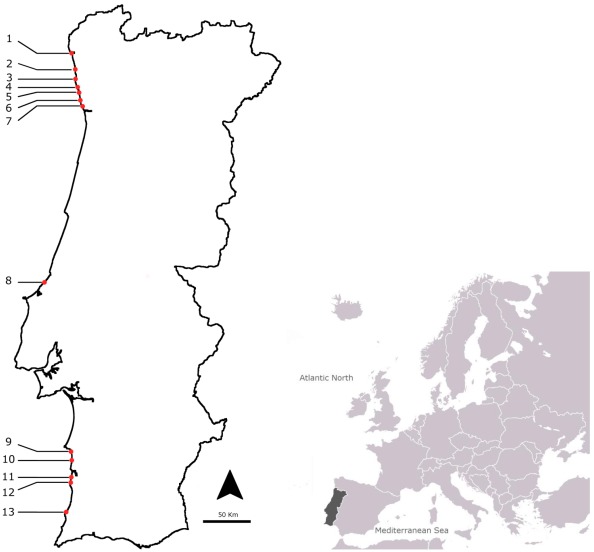
Location of the sampling points in the North Atlantic Portuguese coast: 1 Viana do Castelo; 2 Esposende; 3 Póvoa do Varzim; 4 Angeiras; 5 Memória; 6 Valadares; 7 Aguda; 8 São Martinho do Porto; 9 São Torpes; 10 Porto Côvo; 11 Monte Clérigos; 12 Vila Nova de Milfontes; 13 Almograve.

**Figure 2:**
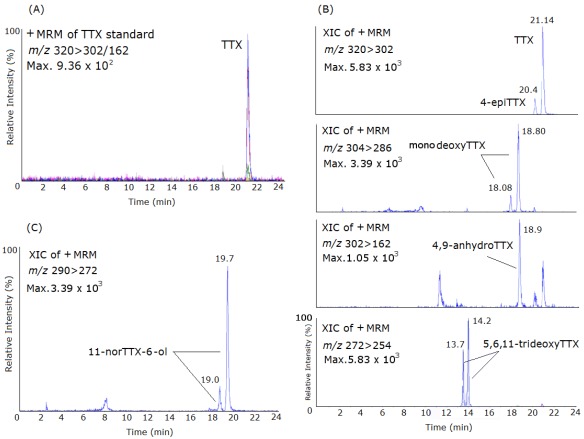
Mass chromatograms of the LC-ESI-CID-MS/MS obtained under MRM operation of the TTX standard and naturally-contaminated samples of *Charonia lampas* and *Lagocephalus sceleratus*. (**A**) MRM of TTX standard (1500 ng/mL), *m/z* 320 > 302; (**B**) Extracted Ion Chromatogram (XIC) of a naturally-contaminated sample of *C. lampas* with TTX and the analogues 4-*epi*TTX; 5,6,11-trideoxyTTX; monodeoxyTTX; 4,9-anhydroTTX; (**C**) XIC of a naturally-contaminated sample of *L. sceleratus* with the analogues 11-norTTX-6-ol *m/z* 290 > 272.

In this study, 134 samples were collected in a monthly sampling program, from July 2009 until November 2010, in 13 sites distributed along the Portuguese coast (Figure 1). The collected species belonged to different taxa and included gastropods (*Monodonta lineata*, *Monodonta turbinata*, *Gibbula umbilicalis*, *Gibbula magus*, *Littorina littorea*, *Littorina saxatilis*, *Nucella lapillus*, *Ocenebra erinacea*, *Calliostoma zizyphinum*, *Patella intermedia*, *Charonia lampas*), bivalves (*Mytilus galloprovincialis*), sea-urchins (*Paracentrotus lividus*) and sea-stars (*Marthasterias glacialis*). The naturally contaminated *Charonia lampas* and *Lagocephalus sceleratus*, obtained in former works [21,24], were used as standards in the LC-MS/MS analysis and provided us with the retention times of TTX and of the other analogues as follows; TTX (21.14 min), 4-*epi*TTX (20.4 min), 5,6,11-trideoxyTTX (13.7–14.2 min), monodeoxyTTX (18.08–18.80 min), 11-norTTX-6-ol (19.0–19.7 min) and 4-anhydroTTX (18.9 min) (Figure 2).

Five protonated molecules [M + H]^+^ at *m/z* 320, 302, 304, 290 and 272 corresponding to TTX plus its derivatives were detected. The MRM transitions selected were: TTX and 4-*epi*TTX: 320 > 302/162; 4,9-anhydroTTX: 302 > 256/162; monodeoxyTTX: 304 > 286/176; 11-norTTX-6-ol: 290 > 272/162 and 5,6,11-trideoxyTTX: 272 > 254/162. Quantification was done with the most abundant ion in the fragment spectra: 302 (TTX and 4-*epi*TTX), 162 (4,9-anhydroTTX), 286 (monodeoxyTTX), 272 (11-norTTX-6-ol) and 254 (5,6,11-trideoxyTTX) (Figure 2).

The limits of detection and quantification (LOD/LOQ) of the LC-ESI-CID-MS/MS for TTX were 16 ng/mL(S/N > 3) and 63 ng/mL (S/N > 10), respectively. 

**Figure 3:**
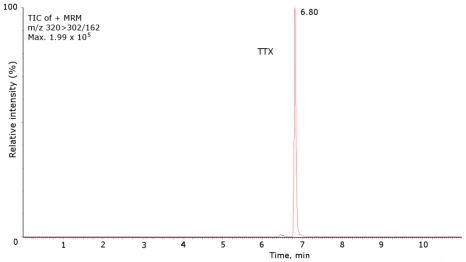
Mass chromatograms of the UPLC-MS/MS obtained under MRM operation of the TTX standard. TIC (total ion chromatogram). TTX standard 1000 ng/mL, *m/z* 320 > 302/162.

For the UPLC-MS/MS the LOD (S/N > 3) was 1.7 ng/mL, and the LOQ (S/N > 10) was 5 ng/mL. TTX and analogue contents were identified and calculated against TTX standard (Figure 3), presuming that the toxin and its derivatives had the same molar response factor in each apparatus. A sample was considered positive when the toxin levels detected were above the LOQ.

**Figure 4:**
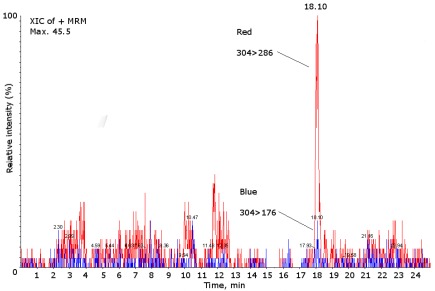
Mass chromatograms of the LC-ESI-CID-MS/MS obtained under MRM operation of the positive sample of *Gibbula umbilicalis *for the analogue monodeoxyTTX (*m/z* 304 > 286/176). XIC extracted ions chromatogram.

The analysis of the gastropod collected in our waters allowed us to detect some positive samples, including *G. umbilicalis*, collected on Memória beach in July 2009. The peaks corresponded to monodeoxyTTX, with an amount of 63.81 ng/g (Figure 4).

**Figure 5:**
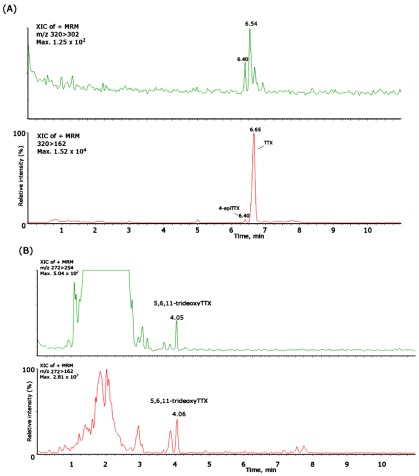
Mass chromatograms of the UPLC-MS/MS obtained under MRM operation of the positive samples of *Monodonta lineata* and *C. lampas* for (**A**): TTX (*m/z *320 > 162/302), 4-*epi*TTX (*m/z *320 > 162/302); and (**B**): 5,6,11-trideoxyTTX (*m/z *272 > 254/162).

Two other gastropod species had positive results: *M. lineata* collected in April 2010 in Vila Nova de Milfontes and *C. lampas* collected in September 2010 in Angeiras (Figure 5). While the first revealed the co-occurrence of TTX (90.50 ng/g) and 4-*epi*TTX (21.48 ng/g), in *C. lampas* we detected low levels of 5,6,11-trideoxyTTX (6.22 ng/g). This last variant was detected previously in the same species, together with TTX [21]. Nevertheless, this is the first report on the occurrence of TTX and variants in the two small gastropods *M. lineata *and *G. umbilicalis. *These two species, regardless of their small size, are harvested and consumed by locals, being rarely found in markets. Thus, the exposure of TTX and analogues via these small gastropods is neither regulated nor controlled. 

The levels of these toxins found in our samples are relatively low when compared to other species that had caused human intoxications, or even with the *C. lampas* that caused the intoxication episode in Malaga [21]. In Table 1 the toxin levels detected in the present work and examples of other works for comparison are displayed. Nevertheless, one should be careful when comparing data, since the levels we report are due to the toxin content in the whole animal (edible part), while the data on the *C. lampas* reported by Rodriguez *et al*. 2008 refers to levels in the digestive gland only [21]. 

**Table 1:** TTX and analogues levels (µg/g) in marine gastropods from Portugal (pw-present work and [21]), China and Taiwan.

Species	Location	TTX	4- *epi*TTX	MonodeoxyTTX	5,6,11-trideoxyTTX	Ref.
*G. umbilicalis*	Memória			0.063		pw
*M. lineata*	Vila Nova de Milfontes	0.090	0.021			pw
*C. lampas*	Angeiras				0.006	pw
	Algarve	315.00 *			1004.00 *	[21]
*N. nitidus*	China	1350				[25]
*N. semiplicatus*		26.10	3.37			[12]
*N. papillosus*	Taiwan	42–60				[26]

* Data obtained from digestive gland only.

The low levels we have found so far may also be due to the fact that TTX synthesis in warmer waters is higher than in the cold North Atlantic ones. 

The “Lessepsian migration” phenomenon may play an important role in the migration of TTX-bearers to more temperate waters, such as the elongated puffer *Lagocephalus sceleratus*, since 1902 more than 62 Red Sea fish species have migrated via the Suez Canal to the Mediterranean [22]. Global warming may also have influenced the migration and settling of TTX-bearing species to more temperate waters, once the rise in water temperature facilitates the migration of Red Sea exotic species to the Mediterranean Sea. Since 5.7% of the Mediterranean fauna is composed of Red Sea fish species, the risk of future invasion by these alien species is potentially very high [22]. There is not yet enough epidemiological and toxicological data concerning human intoxications by TTX in the temperate waters of the Atlantic Ocean. Nevertheless, a study done in the South Pacific area concerning the possible association of ciguatera and climate change revealed interesting assumptions that may give us some hints for future studies [27]. The study projected the idea that a rise in temperature is expected to increase the incidence of ciguatera poisoning of 35–70 per thousand people in 1990 to 160–430 per thousand people in 2050 in Papua New Guinea [27]. Taking into account that we need to be cautious when using simplistic models such as these, the rise in temperature may alter the growth rate of the toxic organisms [28] and also the rates of toxin production [29]. Nevertheless, temperature may also alter accumulation, metabolism and detoxication kinetics in fish vectors as it can for TTX [30]. In fact, another work showed that the *in vitro *uptake of TTX into liver tissue slices of *Takifugo rubripes *is temperature dependent, being significantly higher at 20 °C compared to 5 °C [30]. 

Cellular and molecular studies on the TTX kinetics are also needed in order to better predict the potential effects of global warming on TTX levels in vector species. Taking into account that the microflora of many puffer fish may be the origin of TTX, studies on the TTX production by the main bacterial species are important. A study on the diversity of bacteria isolated from the skin, gill and intestine of *Fugo niphobles *showed that temperature may have an effect on the diversity and density of some bacteria [31]. The identification of *Vibrio *sp. species, and in particular of *V. alginolyticus*, was achieved when fish were exposed at 20 and 29 °C but not when reared at 10 °C. In laboratory culture, all strains of this species grew at 20 to 37 °C but very few grew at 10 °C, suggesting a preference for higher temperatures [31]. So, water temperature may have an impact on the growth rate of TTX producing bacteria, being responsible for higher bacteria counts in fish exposed to higher temperatures. More laboratory experiments are needed to support this hypothesis. All these circumstances, together with the fact that TTX travels along the food-chain [5,6,7,8], may favor the establishment of TTX in Atlantic temperate waters. 

In this work, TTX plus its derivatives were detected in the Portuguese coast, not only in species already reported as TTX-bearers (*Charonia lampas*) but also in indigenous species not yet assigned, this being the first report of TTX presence in *Gibbula umbilicalis* and *Monodonta lineata*. Apart from the quantities detected in these animals, there is an imminent danger to the human population, since the toxin travels in the food-chain and it is unknown whether or not there is biomagnification of TTX [5,6,7,8]. This increases the potential danger, because all the species reported are edible and the toxin is water soluble and thermostable [32,33].

The species *Gibbula umbilicalis *and *Monodonta lineata *belong to the same family, Trochidae [34], and in this study we confirm their ability to accumulate TTX. Possibly there is a potential adaptation that is common for both. In addition, TTX was not detected in all specimens belonging to these two species, which could be due to different strains having different capabilities of adaptation. We can make no inferences about the seasonal intake of the toxin, due to the fact that we only had three positives (2.24% of the total sampling), although all of them were in the warmer months.

TTX is present in Portuguese waters, Angeiras being the most northern point of a TTX report in Atlantic temperate waters. Nevertheless, the low concentrations detected are not sufficient to cause a fatal outcome, since the Minimum Lethal Dose for humans is 2 mg [35]. Surveillance is advisable to avoid poisoning incidents and to understand the progress of this emergent phenomenon. 

## 3. Experimental Section

### 3.1. Sampling Points and Selected Species

TTX was reported in gastropods and sea-stars in many parts of the world [5,6,7,8,21,36,37,38,39,40,41,42,43]. Due to this fact and also because we were searching for potential new vectors, fourteen benthic species were selected, belonging to gastropods (*Monodonta lineata*, *Monodonta turbinata*, *Gibbula umbilicalis*, *Gibbula magus*, *Littorina littorea*, *Littorina saxatilis*, *Nucella lapillus*, *Ocenebra erinacea*, *Calliostoma zizyphinum*, *Patella intermedia*, *Charonia lampas*), bivalves (*Mytilus galloprovincialis*), sea-urchins (*Paracentrotus lividus*) and sea-stars (*Marthasterias glacialis*). Samples were collected monthly at various sampling sites distributed along the coast of continental Portugal (Figure 1): Almograve (37°39'11.52"N; 8°48'09.18"W), Vila Nova de Milfontes (37°43'02.19"N; 8°47'34.40"W), Monte Clérigos (37°20'06.92"N; 8°50'48.09"W), Porto Côvo (37°53'33.19"N; 8°47'38.25"W), São Torpes (37°58'53.56"N; 8°47'45.58"W), São Martinho do Porto (39°30'18.29"N; 9°08'18.07"W), Aguda (41°02'52.13"N; 8°39'13.19"W), Valadares (41°5'29.76"N; 8°39'27.05"W), Memória (41°13'50.96"N; 8°43'18.09"W), Angeiras (41°15'50.01"N; 8°43'37.14"W), Póvoa do Varzim (41°22'41.61"N; 8°46'7.39"W), Esposende (41°29'5.19"N; 8°46'45.76"W) and Viana do Castelo (41°41'35.38"N; 8°50'56.70"W). *Charonia lampas* were purchased at local fish markets, being caught along the Angeiras coast (41°15'49.06"N; 8°43'48.43"W). Organisms were collected in the intertidal area during low tide and were transported to the laboratory and refrigerated as soon as possible. Whenever they were not processed immediately, they were frozen at −20 °C. The number of samples collected and the average number of specimens needed to set a composed sample are displayed in Table 2.

**Table 2:** Average number of specimens to set a composed sample and number of samples collected since July 2009 until the end of 2010. Availability of animals is dependent on their geographical distribution and ecology.

Species	Number of Samples Collected from July 2009 till End 2010	Average Number of Animals Collected to Set a Composed Sample
*Gibbula umbilicalis*	34	100
*Gibbula magus*	1	90
*Monodonta lineata*	20	86
*Monodonta turbinata*	21	86
*Nucella lapillus*	13	15
*Littorina littorea*	2	10
*Littorina saxatilis*	4	15
*Ocenebra erinacea*	4	4
*Calliostoma zizyphinum*	1	1
*Patella intermedia*	4	15
*Charonia lampas*	5	1
*Mytilus galloprovincialis*	7	30
*Paracentrotus lividus*	10	10
*Marthasterias glacialis*	8	1

### 3.2. Sample Treatment

Due to the small size of some species, and in order to have enough biomass for the extraction procedure (1 g), all animals were treated in groups with the exception of *Charonia lampas*, *Calliostoma zizyphinum *and *Marthastherias glacialis*. In Table 2 the number of animals needed to set a composed extractable sample is displayed. Samples were extracted based on the methods of Shoji *et al*. and Ito *et al. *with appropriate amendments to the type of sample [44,45] as follows. Gastropods were boiled in water for 30 min due to their hard consistency. All samples were dissolved in acetic acid (1%), then homogenized with a mechanical crusher (1000 rpm, 5 min), (Silentcrusher M, Heidolph, VWR, Carnaxide, Portugal), and ultrasonication (70 Hz, 3 min), (Vibra Cell, Sonic & Materials, Reagent 5, Porto, Portugal). The extracts were then centrifuged at 4495 g for 20 min (Centrifugal-Legend RT, Sorvall). This procedure was repeated twice, and the two supernatants were defatted with dichloromethane (v/v). Both layers were collected and concentrated under reduced pressure (40 °C) in a rotary evaporator (Büchi) and stored at −20 °C [44,45,46,47,48]. This method was used in the first 50 samples, and then the extraction method was optimized in order to obtain cleaner extracts, based on the protocols of Jen *et al*. and Tsai *et al.* [49,50]: 1 g of sample tissue was homogenized with a blender (A320R1, 700W, Moulinex) then extracted in 3 mL of acetic acid (1%)/methanol with the help of a vortex mixer for 5 min (Top Mix 1118, Fisher Bioblock Scientific) and ultrasonic bath, (5 min, 100 Hz) (RK100H, Bandelin SONOREX). A double extraction was performed, extracts were centrifuged at 4495 g for 15 min at 4 °C (Centrifugal-Legend RT, Sorvall), supernatants were combined and adjusted to a final volume of 7 mL. Then 1 mL of the extract was cleaned through a C18 solid-phase extraction (SPE) cartridge (500 mg/3 mL volume from Supelco, Bellefonte, PA, USA). The cartridges were previously conditioned with 6 mL of methanol, followed by 6 mL of water (milliQ). The sample was eluted with 10 mL of 100% methanol and diluted with the same solvent to a final volume of 12 mL. Finally, each sample was concentrated by drying and re-suspended in 1 mL of methanol, and 100 µL were filtered through 0.45 µm filters (UltraFree-MC centrifugal devices, Millipore, Spain) before LC-MS/MS analysis [49,50]. All reagents used were paranalysis grade from Merck^®^.

### 3.3. LC-MS/MS Analysis

The LC-MS conditions were the same as reported by Rodriguez *et al*. [24]. Briefly, the analyses were performed in high-performance liquid chromatography (LC) equipment consisting of a binary system of LC-10ADVP pumps, an autoinjector (SIL-10ADVP) with degasser (DGU-14A), refrigerated rack, column oven (CTO-10ACvp) and a system controller (SCL-10Avp) from Shimadzu (Kyoto, Japan). The LC system was coupled to a 2000 QTRAPLC/MS/MS instrument from Applied Biosystems (Calrsbad, CA, USA), formed by a hybrid quadrupole-linear ion trap mass spectrometer (MS), equipped with an atmospheric pressured ionisation (API) unit, fitted with an electrospray ionisation source (ESI), operating in the conventional mode of low energy of collision dissociation induced (CID) of MS/MS. Nitrogen was produced by a Nitrocraft NC_LC/MS_ generator from Air Liquide (Madrid, Spain).

The LC system operated with the ESI interface in positive ion mode using the following parameters: curtain gas, 15 psi; collision-activated dissociation gas, 6 psi; IonSpray voltage, 4000 V; temperature, 450 °C; gas 1, 50 psi; gas 2, 50 psi; these parameters had been previously optimized using the TTX standard (Calbiochem Corporation). For the equipment control, data processing and analysis, Analyst software was used. Eluent (A) of the mobile phase was composed by formic acid (Merck, Madrid, Spain) and ammonium formate (Sigma Aldrich, Madrid, Spain), both with a concentration of 10 mM in water, and eluent (B) consisted of acetonitrile (Panreac Quimica, Barcelona, Spain) in water (95:5) with ammonium formate (5 mM) and formic acid (2 mM). The gradient used started with 100% of mobile phase (B), decreasing to 65% at minute 15, rising to 100% at minute 18 until the end of the run (25 min). An XBridge^TM^ Amide column (i.d. 2.1 × 150 mm; 3.5 µm) with a guard cartridge (i.d. 2.1 × 10 mm) from Waters (Cerdanyola del Vallès, Spain) was used to achieve the separation of TTX, and analogues present in the samples. Column oven temperature was set at 25 °C and injection volume was 5 µL. The MS was operated in multiple reactions monitoring (MRM) mode, analysing two product ions per compound: one for quantification and the other for confirmation.

The mass spectrometer parameters were adjusted to obtain a signal of maximum intensity and stability. For the MS optimization, the sample solution was directly infused in the electrospray source at a 0.2 mL/min flow rate with a syringe pump. The MS was operated in the positive ion mode using the product ion scan with a cone gas, 40 V; capillary voltage, 2.8 kV; source temperature, 120 °C; desolvation temperature, 350 °C; collision energy, 45 eV. Helium and nitrogen were used as collision and drying gases, respectively [24]. 

To overcome the challenge of the lack of standards for TTX analogues [44,51,52,53,54,55] a sample of a naturally-contaminated *Charonia lampas* and *Lagocephalus sceleratus* used in former works [21,24] was injected in the LC-MS/MS, this way determining the respective retention times (RT) (Figure 2). The following variants were aimed for: TTX, 4-*epi*TTX, 5,6,11-trideoxyTTX, monodeoxyTTX, 11-norTTX-6-ol and 4-anhydroTTX. For the calibration curve, several dilutions from the TTX standard were performed, from 50 ng/mL concentration to 2000 ng/mL. TTX and its derivatives were quantified, using their peak areas to calculate amounts and using the curve obtained from TTX standard [56]. 

### 3.4. UPLC-MS/MS Analysis

Samples were analysed in Ultra High Performance Liquid Chromatography equipment ACQUITY UPLC system, coupled to a Xevo TQ MS mass spectrometer from Waters (Manchester, UK). The apparatus is equipped with a multimode source ESI/APCI/ESCi, a vacuum system composed of two air-cooled Edwards Vacuum turbo molecular pumps evacuating the source and analyzer, one Varian rotary backing pump. The nitrogen generator was a Nitrocraft NC_LC/MS_ from Air Liquide (Madrid, Spain). Chromatographic separation and detection of TTX and its derivatives was achieved with a Waters Acquity UPLC BEH Amide column (100 mm × 2.1, 1.7 μm), equipped with a 0.2 μm Acquity UPLC in-line filter and column oven at 35 °C. The LC operated with eluent (A), consisting of 10 mM ammonium formate (Sigma Aldrich, Madrid, Spain) and 10 mM formic acid (Merck, Madrid, Spain) in water. Eluent (B) contained acetonitrile (Panreac Quimica, Barcelona, Spain) in water (95:5) with a final concentration of 5 mM ammonium formate and 2 mM formic acid. The gradient programme used to elute the toxins was 100% mobile phase (B) at the beginning, decreasing to 65% (B) after 7 min, then kept for 2 min and back to 100% (B) over the next 0.5 min and finally kept 100% (B) for 1.5 min before the next injection. Flow rate was 0.4 mL/min and injection volume was 5 µL.

The Xevo TQ MS mass spectrometer operated with the following optimized source-dependent parameters (ESI source): capillary potential 2.7 kV, cone voltage 40 V, desolvation temperature 350 °C, desolvation gas flow 850 L/h N_2_, cone gas flow 50 L/h N_2_, source temperature 150 °C, collision gas flow 20 V. Argon was used as the collision gas at 4.5 × 10^−3^ mbar.

The mass spectrometer operated in MRM, detecting in positive mode, analysing two product ions per compound: one for quantification and another for confirmation. The transitions employed were: TTX and 4-*epi*TTX (*m/z* 320 > 302/162) and 5,6,11-trideoxyTTX (272 > 254/162), with retention times: TTX (6,80 min), 4-*epi*TTX (6.50 min) and 5,6,11-trideoxyTTX (4.06 min). Quantification was undertaken with the most abundant ion in the fragment spectra: 162 for TTX, 4-*epi*TTX and 5,6,11-trideoxyTTX. TTX analogues in sample solutions were identified according to the daughter ion spectra of the analogues reported in the literature [44]. For the calibration curve, several dilutions from the TTX standard (Calbiochem Corporation) were performed, from 31.25 ng/mL concentration to 3000 ng/mL. TTX and its derivatives were quantified using their peak areas to calculate amounts and using the curve obtained from TTX standard [56]. 

## 4. Conclusions

In this work we used LC-MS/MS and UPLC-MS/MS to detect TTX and several analogues in three autochthonous gastropod species of the Atlantic Portuguese continental coast. TTX, 4-*epi*TTX, monodeoxyTTX and 5,6,11-trideoxyTTX were detected in *Monodonta lineata*, *Gibbula umbilicalis *and *Charonia lampas*, being the most northern point of the Atlantic Ocean were these toxins were reported. All these species are edible, raising the probability of human health hazards. Despite the low concentrations detected, ranging from 6.22 to 90.50 ng/g, it was clearly shown that TTX and analogues should be monitored in the species reported positive, and in others that can potentially accumulate the toxins and can be used as human food. 
